# Probiotics and Postbiotics for Green Control of Foodborne Pathogens: Intelligent Detection and Biopreservation Strategies for Safer Foods

**DOI:** 10.3390/foods14183281

**Published:** 2025-09-22

**Authors:** Alice N. Mafe, Dietrich Büsselberg

**Affiliations:** 1 Department of Biological Sciences, Faculty of Sciences, Taraba State University, Main Campus, Jalingo 660101, Taraba State, Nigeria; mafealice1991@gmail.com; 2 Department of Physiology and Biophysics, Faculty of Medicine, Weill Cornell Medicine-Qatar, Education City, Qatar Foundation, Doha Metropolitan Area, Doha P.O. Box 22104, Qatar

**Keywords:** AIEE-type Supra-CDs, p-PA, TU, controllable IFE, probiotics, postbiotics, biopreservation, food safety, sustainable preservation

## Abstract

The extensive use of chemical preservatives in the food industry has raised concerns over their association with gut microbiota imbalance, allergenic reactions, and potential carcinogenicity. Growing consumer demand for “clean label” products, coupled with regulatory pressures, has accelerated the search for safer and more sustainable alternatives. In this study, it is reported for the first time that the synthesis of AIEE-type Supra-CDs using p-phenylenediamine (p-PA) and thiourea (TU), a breakthrough that provides a new class of nanomaterials with superior optical and antimicrobial properties. More importantly, the study demonstrates a quantitative improvement of spectral overlap through controllable inner filter effect (IFE), establishing a reliable strategy to enhance detection sensitivity and broaden applicability in food safety monitoring. Beyond their intrinsic antimicrobial potential, these Supra-CDs integrate seamlessly with intelligent detection platforms such as biosensors, CRISPR-based assays, and AI-assisted analytics, enabling real-time evaluation of probiotic- and postbiotic-based preservation systems. By combining novel material synthesis with precision monitoring technologies, this work offers a dual innovation: reducing reliance on synthetic additives while providing scalable tools for sustainable food preservation. The findings not only advance the frontier of biopreservation research but also align with global initiatives for consumer health and environmental sustainability.

## 1. Introduction

In recent years, consumer demand for minimally processed foods free from synthetic additives has risen sharply, reflecting growing awareness of health, nutrition, and sustainability [1]. Although chemical preservatives remain widely used in the food industry due to their effectiveness in prolonging shelf life, increasing evidence has linked their consumption to potential health risks, including gut dysbiosis, allergic reactions, and carcinogenic effects [2]. These concerns have intensified the search for safer, natural alternatives that meet both consumer expectations and regulatory requirements for “clean label” products [3]. Probiotics and postbiotics have gained attention as promising biopreservation agents due to their dual role in promoting health and exerting antimicrobial activity [4]. Probiotic microorganisms, particularly lactic acid bacteria such as *Lactobacillus* sp. and *Bifidobacterium* sp., suppress the growth of pathogens by producing inhibitory metabolites and competing for nutrients and ecological niches [5]. Their non-viable derivatives, collectively known as postbiotics, include organic acids, bacteriocins, and peptides that retain stability and functionality while offering potent antimicrobial properties [6]. In view of these advantages, the adoption of these as natural preservatives in mainstream food production remains limited.

A critical research gap lies in the lack of integrated studies that connect the antimicrobial mechanisms of probiotics and postbiotics with modern monitoring technologies [7]. Emerging tools such as biosensors, omics-based biomarkers, CRISPR-based detection systems, and artificial intelligence models can provide real-time insights into microbial activity and food safety [8]. However, their potential synergy with biopreservation strategies has not yet been fully explored or systematically reviewed [9].

Recent progress in nanomaterial-based detection technologies, particularly aggregation-induced emission enhancement (AIEE)-type carbon dots, has further expanded the possibilities for food safety monitoring [10]. These materials exhibit tunable photophysical properties, high stability, and controllable inner filter effects (IFE), making them excellent candidates for sensitive detection platforms [11]. Representative studies from 2023 to 2024 have demonstrated how regulating the AIEE mechanism in carbon dots can improve spectral overlap, enhance signal-to-noise ratios, and broaden applicability in complex food matrices [12]. For example, a lychee-like plasmonic nanocomplex with a programmable hierarchical structure was recently developed as a high-performance SERS platform for monitoring diazepam in aquatic products, showcasing how novel nanostructures can deliver superior sensitivity and selectivity [13].

This review aims to address the existing gap by evaluating the application of probiotics and postbiotics in controlling foodborne pathogens, while also examining advancements in intelligent detection platforms. By integrating insights from AIEE-type carbon dots and related nanotechnologies, it further underscores the convergence of natural biopreservation with next-generation monitoring strategies. In doing so, it aims to highlight pathways for safer, sustainable, and scalable solutions to food preservation in industrial practice.

## 2. Chemical Preservatives in Food Safety: Risks and Limitations

### 2.1. Common Chemical Preservatives and Their Uses

Chemical preservatives remain a cornerstone of food preservation strategies, valued for their ability to extend shelf life and reduce microbial spoilage [14]. Widely used compounds include nitrites, benzoates, sorbates, sulfites, and parabens, each of which is selected for specific applications [15]. Nitrites, for example, are added to cured meats to inhibit *Clostridium botulinum*, while benzoates and sorbates are incorporated into acidic foods and beverages to control yeast and mold growth [16]. Sulfites are commonly found in dried fruits, wines, and certain processed foods, where they help maintain color and prevent oxidative changes [17]. These substances are attractive to food manufacturers because they are inexpensive, relatively easy to apply, and effective against a wide range of microorganisms. As summarized in Table 1, chemical preservatives remain widely applied across food systems, although their potential health concerns warrant scrutiny.

### 2.2. Documented Health Risks

Given their usefulness, the reliance on synthetic preservatives is not without drawbacks. Several studies have raised concerns regarding their potential adverse health effects. Nitrites and nitrates can give rise to nitrosamines, which are recognized carcinogens [27]. Sulfites are well known to provoke allergic reactions, particularly in individuals with asthma, while benzoates in combination with artificial colors have been associated with hyperactivity in children [28]. Parabens and some synthetic antioxidants have also come under scrutiny due to possible endocrine-disrupting properties [29]. Beyond these direct effects, many preservatives may negatively alter the gut microbiota, leading to dysbiosis and long-term health implications. Although regulatory bodies have established acceptable daily intake levels, uncertainties remain about the cumulative and chronic impacts of regular exposure.

### 2.3. Consumer-Driven Demand for Alternatives

Alongside scientific concerns, consumer preferences are evolving rapidly. Modern consumers increasingly prioritize foods labeled as “natural,” “organic,” or “free from artificial additives,” reflecting broader awareness of wellness and sustainability [30]. This shift is driving both market and regulatory interest in natural preservation strategies. Biopreservation, particularly through probiotics, postbiotics, and plant-derived antimicrobials, is gaining attention as a viable alternative [31]. These natural systems not only provide antimicrobial protection but also offer added health benefits, aligning firmly with the global movement toward clean-label foods.

## 3. Probiotics and Postbiotics in the Context of Food Safety

### 3.1. Definitions and Differences

Probiotics are beneficial live microorganisms that, when consumed in sufficient quantities, provide health benefits to the host [32]. Within food preservation, they are recognized not only for supporting gut health but also for their antimicrobial effects against spoilage organisms and pathogens [33]. Postbiotics, on the other hand, are defined as the inactivated microbial cells, their structural components, or their metabolites that retain biological activity even in the absence of live organisms [34]. The key distinction lies in functionality: probiotics require viability to exert their benefits, whereas postbiotics act through stable bioactive compounds such as organic acids, peptides, and bacteriocins [35]. This makes postbiotics particularly attractive in food safety, as they bypass challenges associated with maintaining probiotic survival during processing, storage, or gastrointestinal passage.

### 3.2. Key Microbial Strains and Metabolites

Lactic acid bacteria (LAB) remain the most studied group of microbes with biopreservation potential [36]. Species from *Lactobacillus* sp., *Bifidobacterium* sp., and *Pediococcus* sp. produce an array of antimicrobial substances, including lactic and acetic acids, hydrogen peroxide, diacetyl, and ethanol, which lower pH and inhibit pathogen growth [37]. In addition, peptide-based antimicrobials such as nisin, pediocin, and reuterin demonstrate strong activity against Gram-positive bacteria like *Listeria monocytogenes* and *Staphylococcus aureus* [38]. Beyond LAB, specific yeasts and fungi also generate antimicrobial compounds that may serve as effective postbiotics [39]. Collectively, these metabolites form a natural arsenal that enhances food safety while extending shelf life. As shown in Table 2, different probiotic species generate metabolites with promising preservative potential.

### 3.3. Mechanistic Pathways in Inhibiting Pathogens

**Figure 1:**
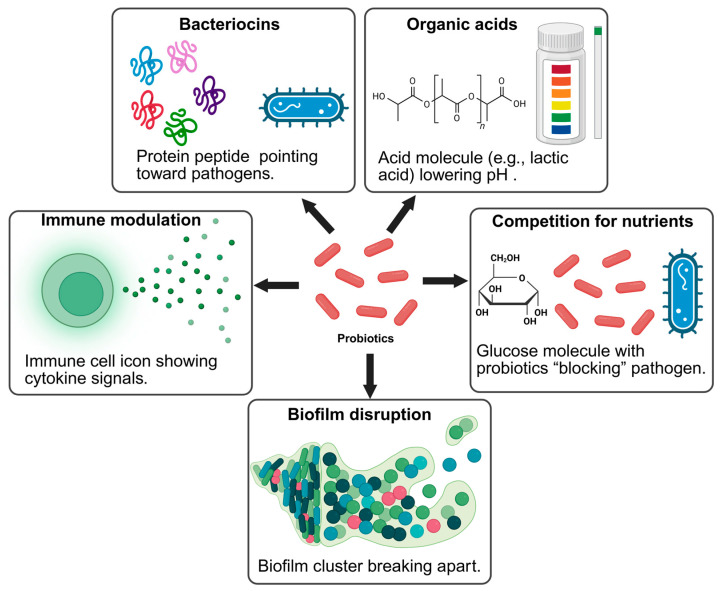
Mechanisms by Which Probiotics and Postbiotics Inhibit Foodborne Pathogens (created with BioRender). Dietrich B. (2025) https://app.biorender.com/illustrations/68b2e1f158b56a70a2c6caf7 (accessed on 29 August 2025).

The antimicrobial actions of probiotics and postbiotics are driven by diverse mechanisms. Organic acid production reduces environmental pH, destabilizing pathogenic cells, while hydrogen peroxide exerts oxidative stress on susceptible organisms [50]. Bacteriocins and antimicrobial peptides disrupt bacterial membranes by forming pores, which leads to leakage of intracellular material and eventual cell lysis [51]. Probiotic strains can also compete with pathogens for adhesion sites and nutrients, thereby limiting colonization opportunities [52]. Additionally, some probiotics interfere with quorum-sensing systems, disrupting pathogen communication, virulence expression, and biofilm formation [53]. These combined mechanisms underscore the effectiveness of probiotic and postbiotic strategies as natural alternatives to chemical preservatives in food safety management. A graphical overview of probiotic and postbiotic actions against foodborne pathogens is presented in Figure 1.

## 4. Mechanisms of Biopreservation Against Foodborne Pathogens

### 4.1. Antibacterial Effects: Bacteriocins, Organic Acids, and Hydrogen Peroxide

A central mode of action in biopreservation is the antibacterial activity exerted by probiotics and their metabolites [54]. Lactic acid bacteria (LAB) generate organic acids such as lactic and acetic acids, which reduce the pH of the food matrix and create unfavorable conditions for pathogens [55]. These acids can also permeate bacterial cell membranes, leading to internal acidification and metabolic disruption [56]. Certain strains additionally produce hydrogen peroxide, which induces oxidative stress that damages nucleic acids, proteins, and cell membranes [57]. Bacteriocins are small, ribosomally synthesized antimicrobial peptides such as nisin, pediocin, and reuterin, which further strengthen these effects. They act by binding to bacterial membranes, forming pores, and causing leakage of vital cellular contents, ultimately resulting in cell death [58]. Such compounds are among the most potent natural candidates to replace synthetic preservatives.

### 4.2. Antifungal Activity

Beyond their antibacterial functions, probiotics and postbiotics are effective against spoilage fungi. Metabolites like phenyllactic acid, fatty acids, and specific cyclic peptides have demonstrated inhibitory activity against species of *Aspergillus* sp., *Penicillium* sp., and *Fusarium* sp. [59]. These substances compromise fungal cell wall integrity, disrupt membrane permeability, and interfere with enzymatic pathways necessary for spore germination and hyphal growth [60]. This antifungal capability is instrumental in prolonging the freshness of baked goods, dairy items, and fruit-based foods, where mold contamination is a common challenge [61].

### 4.3. Biofilm Disruption and Quorum Sensing Inhibition

Foodborne pathogens often persist in the form of biofilms, which are protective microbial communities embedded in extracellular polymeric substances [62], which enhance their resistance to cleaning and antimicrobial treatments [63]. Probiotics counter this survival strategy by secreting enzymes, biosurfactants, and bacteriocins that degrade biofilm structures [64]. In addition, some probiotic metabolites can block quorum sensing, the signaling mechanism bacteria use to regulate virulence, toxin production [65], and biofilm development [66]. By disrupting these communication systems, probiotics reduce the pathogenic potential of harmful microbes and improve the microbial safety of foods.

### 4.4. Antioxidant Effects

Another valuable contribution of probiotics and postbiotics is their antioxidant capacity. Through the release of compounds such as exopolysaccharides, peptides, and phenolic derivatives, they scavenge reactive oxygen species and limit oxidative stress within foods [67]. This process slows lipid oxidation and protein degradation, which are major causes of quality loss in perishable products like meats and dairy [68]. Antioxidant activity thus not only protects nutritional value but also preserves sensory characteristics such as flavor, aroma, and color, while reducing the formation of harmful by-products [69].

### 4.5. Case Examples in Dairy, Meat, and Fresh Produce

**Figure 2:**
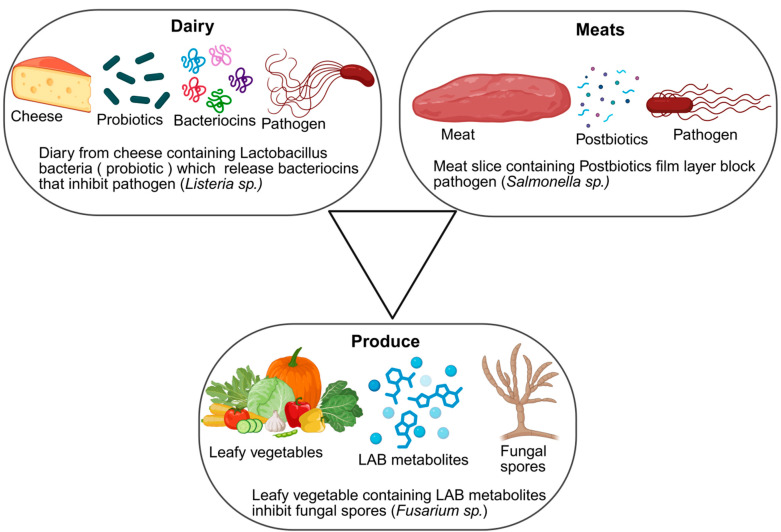
Examples of Biopreservation Mechanisms Across Food Systems (Created with BioRender). Dietrich B. (2025) https://app.biorender.com/illustrations/68b2f65ab205da4cc2df51da (accessed on 29 August 2025).

The practical benefits of these mechanisms are already being demonstrated in various food sectors. In fermented dairy products, *Lactobacillus* strains are used to inhibit *Listeria monocytogenes* while simultaneously enhancing flavor profiles [70]. In meat preservation, bacteriocins such as nisin and pediocin effectively suppress *Clostridium* sp. and *Staphylococcus* sp. without negatively impacting texture or taste [71]. For fresh produce, washes and edible coatings enriched with probiotic-derived metabolites have been shown to reduce contamination by *E. coli* and spoilage fungi [72], thereby extending shelf life [73]. Collectively, these applications highlight how biopreservation strategies offer both microbial safety and quality enhancement across different food categories. Figure 2 provides representative cases of how probiotics and postbiotics contribute to food preservation.

## 5. Intelligent Detection and Monitoring Tools

### 5.1. Biosensors (Electrochemical, Aptamer, CRISPR)

Biosensors have emerged as rapid and sensitive tools for detecting foodborne pathogens and contaminants [74]. Electrochemical biosensors rely on measurable electrical signals generated by microbial interactions, making them suitable for real-time monitoring [75]. Aptamer-based biosensors, which utilize short nucleic acid sequences with high affinity for specific targets, provide precision in pathogen recognition and are increasingly used as alternatives to antibodies [76]. CRISPR-based biosensors are a recent innovation, leveraging gene-editing technology to achieve highly specific and ultra-sensitive detection [77]. Together, these platforms offer significant advantages over traditional microbiological methods, including shorter detection times, portability, and potential for on-site applications.

### 5.2. Omics-Based Approaches (Metabolomics, Proteomics, Microbiome Shifts)

Omics technologies have transformed the way food safety risks are evaluated [78]. Metabolomics enables the identification of microbial metabolites that serve as biomarkers for spoilage or pathogenic contamination [79]. Proteomics provides insights into protein expression patterns associated with microbial survival and virulence in different food matrices [80]. Additionally, microbiome analysis helps track shifts in microbial communities during storage, processing, and preservation, revealing early warning signs of contamination [81]. By combining these approaches, researchers and food safety professionals can achieve a systems-level understanding of pathogen behavior and food quality changes, enabling more proactive safety measures.

### 5.3. AI/Machine Learning for Pathogen Risk Prediction

Artificial intelligence (AI) and machine learning are increasingly being applied to predict and prevent food safety hazards. These technologies analyze large datasets from microbial genomics, environmental monitoring, and supply chain records to identify patterns and predict contamination risks before they escalate [82]. For example, AI algorithms can be trained to detect correlations between storage conditions and pathogen growth or to forecast spoilage timelines based on historical data [83]. Machine learning also facilitates adaptive food safety models that continuously improve with new information, enhancing the accuracy and efficiency of risk assessments across diverse food systems.

### 5.4. Integration into Food Production Pipelines

**Figure 3:**
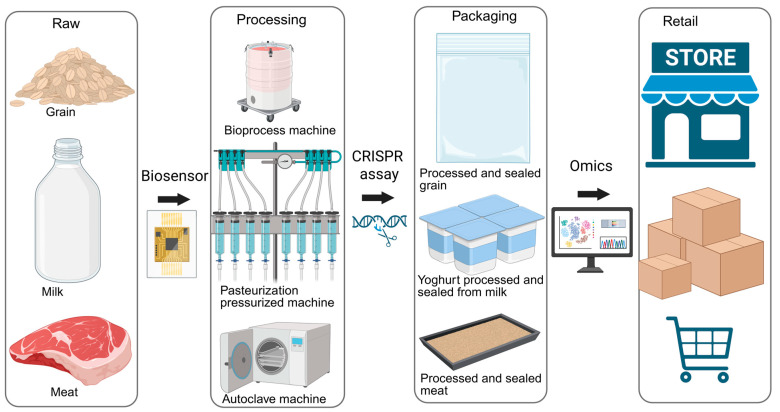
Integration of Intelligent Detection Tools into Food Production Pipelines (created with BioRender). Dietrich B. (2025) https://app.biorender.com/illustrations/68b2f719c899f0aff850d478 (accessed on 29 August 2025).

The integration of intelligent detection and monitoring tools into food production pipelines represents a significant step toward proactive and preventive safety management [84]. Real-time biosensor systems can be embedded in processing lines to detect pathogens instantly, reducing reliance on post-production testing [85]. Omics and AI-based models can be incorporated into quality control frameworks to guide decision-making in storage, packaging, and distribution [86]. Such integration not only minimizes the risk of outbreaks but also aligns with the goals of precision food safety, where interventions are tailored based on real-time data [86]. Ultimately, these technologies enable a shift from reactive to predictive food safety strategies, ensuring both consumer protection and industry efficiency. Figure 3 illustrates the integration of intelligent detection tools into modern food production pipelines.

## 6. Review of Research Findings & Applications

### 6.1. Evidence from Bench to Industry

Over the past two decades, research on biopreservation has steadily transitioned from laboratory investigations to industrial-scale applications [87]. Early bench studies focused on isolating specific antimicrobial compounds such as bacteriocins, organic acids, and postbiotic metabolites, demonstrating their inhibitory activity against major foodborne pathogens [88]. These findings provided the foundation for scaling up processes, optimizing formulations, and integrating them into food manufacturing systems. Today, several biopreservation strategies have been successfully tested in pilot plants and commercial settings, reinforcing their potential as practical and sustainable solutions for food safety.

### 6.2. Case Studies: Dairy, Meats, Beverages, Plant-Based Foods

Applications of biopreservation vary widely across food categories [89]. In the dairy industry, bacteriocin-producing lactic acid bacteria have been utilized to control *Listeria monocytogenes* in cheese and yogurt [90]. In meat products, protective cultures and organic acid-releasing biopolymers have extended shelf life while maintaining sensory quality [91]. Fermented beverages benefit from yeast–bacteria interactions that suppress spoilage organisms and stabilize flavor [92]. Similarly, plant-based foods, including fresh produce and meat alternatives, have shown improved microbial stability when treated with natural antimicrobials or coated with probiotic-infused films [93]. These case studies highlight the versatility of biopreservation approaches across diverse food matrices.

However, a recurring limitation across many real sample analyses is the lack of validation against gold-standard analytical methods. For example, while probiotic or postbiotic interventions are reported to enhance preservation, metabolite quantification is often expressed only as relative changes or inhibition zones without cross-checking using robust techniques such as HPLC or LC–MS [94,95]. This can lead to discrepancies and restrict comparability between studies. Future research should therefore prioritize integrating such validation methods to strengthen the reliability, reproducibility, and industrial applicability of biopreservation findings across different food matrices

### 6.3. Regional Evidence (Africa, Asia, EU, USA)

Regional adoption of biopreservation reflects both local food systems and regulatory frameworks [96]. In Africa, research emphasizes the use of indigenous lactic acid bacteria from traditional fermented foods to enhance the safety of milk and cereal-based products [97]. Asian studies highlight applications in seafood and soy-based foods, aligning with high demand for minimally processed products [98]. The European Union has advanced regulatory approval of several bacteriocins and probiotic strains, supported by consumer preference for clean-label foods [99]. In the USA, biopreservation research is often linked to large-scale food safety programs and innovation in the plant-based food sector [100]. Together, these regional perspectives demonstrate both global interest and context-specific innovation. As indicated in Table 3, regional variations strongly influence the adoption of probiotic and postbiotic preservation methods.

### 6.4. Comparison with Chemical Preservatives

When compared to chemical preservatives, biopreservation strategies offer a distinct set of advantages [107]. Unlike synthetic additives, biopreservatives often provide dual benefits, suppressing microbial growth while contributing to gut health, antioxidant activity, or improved sensory attributes [108]. Furthermore, they align with consumer demand for natural, minimally processed foods, reducing reliance on controversial additives such as nitrites, sulfites, and parabens [109]. However, biopreservation may face challenges, including higher production costs, variability in strain performance, and shorter shelf-life stability in some applications [110]. Given these limitations, accumulating evidence indicates that biopreservation represents a safer, consumer-friendly, and sustainable alternative to conventional chemical preservatives.

## 7. Innovations & Technological Advances

### 7.1. Encapsulation for Stability

Encapsulation has emerged as a powerful approach to enhance the stability and efficacy of probiotics and postbiotics in food systems [111]. By embedding these bioactives within protective matrices such as alginate, chitosan, or lipid-based carriers, they are shielded from harsh processing conditions, oxygen, and gastric acidity [112]. This not only prolongs shelf life but also ensures controlled release at targeted sites, thereby maintaining their antimicrobial and health-promoting properties in functional foods.

### 7.2. Edible Coatings & Films Enriched with Postbiotics

The incorporation of postbiotics into edible coatings and biodegradable films represents another frontier in food preservation [113]. These coatings create a physical barrier against microbial contamination while releasing antimicrobial metabolites, such as organic acids or bacteriocins, directly onto the food surface [114]. Applications in fruits, vegetables, and ready-to-eat foods have demonstrated reduced spoilage, extended shelf life, and enhanced food safety, while aligning with sustainability goals [115].

### 7.3. Synergistic Preservation with Essential Oils and Nanomaterials

Combining probiotics or postbiotics with natural antimicrobials such as essential oils and emerging nanomaterials offers synergistic effects that improve food preservation [116]. Essential oils contribute additional antimicrobial and antioxidant activity, while nanomaterials provide enhanced delivery, stability, and surface activity [117]. This multifaceted strategy has shown promise in improving microbial control and oxidative stability across diverse food products, without compromising sensory qualities [118].

### 7.4. Smart Packaging Linking Probiotic/Postbiotic Release and Detection

Smart packaging technologies are now being designed to integrate probiotic or postbiotic release systems with real-time detection tools [119]. Such packaging can respond to environmental triggers like pH changes or microbial growth by releasing protective metabolites while simultaneously signaling spoilage through colorimetric or biosensor-based indicators [120]. This dual function not only enhances food safety but also provides consumers and industries with transparent and intelligent monitoring of product quality.

## 8. Challenges & Limitations

### 8.1. Stability and Viability in Food Matrices

Although probiotics and postbiotics hold great promise for food preservation, their stability within food systems remains a major hurdle [121]. Processing conditions such as high temperature, variable pH, and low water activity can compromise their functional activity [122]. In addition, interactions with other food components may further reduce their effectiveness. While protective technologies, such as encapsulation, have shown encouraging results, ensuring consistent performance across different food types and large-scale production processes remains a considerable challenge. It is also important to note that stability data should clearly report both initial and residual values when assessing probiotic viability or postbiotic metabolite retention [34,123]. Presenting only percentage decreases without baseline values can lead to contradictory interpretations, as the magnitude of loss cannot be accurately contextualized. To improve reliability and comparability across studies, future research should supplement percentage changes with original data, thereby providing a clearer picture of actual stability outcomes in food matrices. A consolidated list of current challenges in probiotic and postbiotic preservation is presented in Table 4.

### 8.2. Regulatory Ambiguity Around “Postbiotics”

A critical limitation to the wider adoption of postbiotics is the absence of clear and harmonized regulatory frameworks [131]. Most national and international food safety authorities have yet to establish whether postbiotics should be treated as additives, bioactive ingredients, or therapeutic agents [132]. This lack of clarity creates obstacles for manufacturers, delays product approvals, and complicates labeling and health claims [133]. Reaching a standardized definition and regulatory consensus will be essential for smoother industrial application and consumer acceptance.

### 8.3. Cost and Scalability

Moving from laboratory-scale innovation to industrial-scale production introduces significant economic challenges [134]. Advanced technologies such as microencapsulation, nanotechnology, or smart packaging often involve high production costs compared to conventional preservatives [135]. Furthermore, achieving reproducibility and maintaining quality during large-scale bioprocessing requires specialized infrastructure and expertise [136]. Without cost-effective and scalable approaches, the widespread use of these bio-based alternatives may remain limited, particularly in developing regions.

### 8.4. Risk of Antimicrobial Resistance

Another concern lies in the potential contribution of microbial metabolites, particularly bacteriocins, to antimicrobial resistance [137]. While they are generally safer than synthetic antibiotics, prolonged or uncontrolled use could still exert selective pressure on microorganisms. Continuous monitoring, careful safety assessments, and regulated application are necessary to minimize this risk [138]. Addressing these issues proactively will be vital for ensuring the safe and sustainable integration of probiotics and postbiotics into food systems.

## 9. Future Directions

### 9.1. Precision Fermentation and Synthetic Biology

Emerging tools in precision fermentation and synthetic biology provide novel avenues for producing postbiotics with higher efficiency, consistency, and specificity [139]. By engineering microbial pathways, it becomes possible to enhance the biosynthesis of desirable metabolites such as peptides, organic acids, and bacteriocins [140]. This approach minimizes variability often observed in traditional fermentation while improving scalability for industrial applications [141]. As these technologies advance, they are expected to reshape the way postbiotics are manufactured for food and health-related uses.

### 9.2. Multi-Strain, Matrix-Specific Formulations

A key future direction lies in designing multi-strain formulations that are tailored to particular food matrices [142]. Different strains of lactic acid bacteria and their metabolites can act synergistically, providing stronger preservative functions and broader functional effects [143]. Formulations optimized for the conditions of dairy, plant-based foods, beverages, and other systems could increase both stability and effectiveness [144]. Such matrix-specific innovations will expand the versatility of postbiotics while enhancing their acceptability across diverse product categories.

### 9.3. Deployment in LMICs Using Local LAB Strains

In low- and middle-income countries (LMICs), the application of indigenous lactic acid bacteria (LAB) strains represents a practical and sustainable solution [145]. Local strains are often better adapted to traditional fermentation processes and raw materials, making them more resilient and cost-effective for use in local food systems [146]. Strengthening their application could help reduce post-harvest losses, improve food safety, and promote food security, while also aligning with cultural practices and dietary habits.

### 9.4. Global Regulatory Harmonization

The rapid growth of postbiotic research and applications highlights the need for globally harmonized regulatory frameworks [147]. Currently, inconsistencies in definitions, safety requirements, and labeling standards across jurisdictions hinder the commercialization and trade of postbiotic products [148]. Establishing unified guidelines will provide clarity to industry stakeholders, enhance consumer confidence, and accelerate the development of safe and effective postbiotic-based solutions in global markets.

### 9.5. Consumer Perception and Acceptance Studies

Ultimately, the success of postbiotic innovations depends on consumer awareness and acceptance [149]. While interest in gut health and natural preservation is increasing, skepticism and limited understanding still pose challenges [150]. Future work should focus on understanding consumer attitudes, preferences, and willingness to adopt such products. Transparent communication, evidence-based claims, and educational campaigns will be essential in fostering trust and driving widespread acceptance of postbiotic-based functional foods.

## 10. Conclusions

Probiotics and postbiotics are emerging as sustainable and multifunctional alternatives to synthetic preservatives, combining antimicrobial effectiveness with added health-promoting properties. Their natural origin makes them attractive within the context of eco-friendly preservation strategies, aligning with the growing global demand for safer and more sustainable food systems. By simultaneously enhancing food safety and nutritional value, they hold strong potential as next-generation preservation tools. The application of advanced detection and monitoring technologies will play a critical role in maximizing the effectiveness of these bioactive systems. Intelligent packaging and biosensing platforms can verify their safety, stability, and functionality, thereby ensuring consistency and regulatory compliance. Such integration of biological innovation with smart validation tools will help build greater confidence among both industry stakeholders and consumers. For these technologies to achieve large-scale impact, progress is required in three main areas: regulatory clarity, industrial scalability, and consumer acceptance. Clear guidelines and harmonized standards will accelerate adoption, while technological advances in bioprocessing can lower costs and enable wider application. At the same time, transparent communication and education will be essential in building trust. With these enablers in place, probiotics and postbiotics can evolve into reliable, sustainable, and consumer-approved food safety solutions for the future.

## Figures and Tables

**Table 1:** Commonly Used Chemical Preservatives, Their Applications, and Associated Health Concerns.

Preservative	Food Application	Mode of Action	Documented Risks
Sodium nitrite/nitrate	Processed meats, sausages, cured fish	Inhibits *Clostridium botulinum* via nitrosylation	Carcinogenic nitrosamine formation; linked to colorectal cancer [18]
Sodium benzoate	Beverages, sauces, jams	pH-dependent inhibition of yeasts, molds, some bacteria	Allergic reactions, hyperactivity in children, DNA damage in vitro [19]
Potassium sorbate	Cheese, bakery products, soft drinks	Inhibits molds and yeasts via disruption of cell membranes	Potential genotoxic effects; mucosal irritation [20]
Sulfites (SO_2_, sodium metabisulfite)	Dried fruits, wine, juices, seafood	Antioxidant and antimicrobial action	Asthma exacerbation, allergic reactions, gut microbiota disruption [21]
Propionic acid and salts	Bakery products, cheeses	Inhibits molds by lowering intracellular pH	Gastrointestinal discomfort; potential microbiome imbalance [22]
Parabens (methyl-, propyl-paraben)	Beverages, sauces, cosmetics	Disrupts microbial membranes	Endocrine disruption, estrogenic activity [23]
Butylated hydroxyanisole (BHA) & butylated hydroxytoluene (BHT)	Oils, cereals, snacks	Antioxidant to prevent lipid oxidation	Tumorigenic in rodents; oxidative stress induction [24]
Hexamethylenetetramine	Fish, caviar, cheese	Converts to formaldehyde in acidic foods, inhibiting bacteria	Formaldehyde toxicity; respiratory irritation [25]
Calcium propionate	Bread, baked goods	Inhibits molds and *Bacillus* spp.	Behavioral effects in sensitive children; GI distress [26]

**Table 2:** Main Probiotic Strains and Postbiotic Metabolites in Food Preservation.

Microbial Strain	Metabolite(s) Produced	Target Pathogen/Spoilage Organism	Food System Application
*Lactobacillus plantarum*	Lactic acid, bacteriocins (plantaricins), hydrogen peroxide	*Listeria monocytogenes*, *E. coli* O157:H7, molds	Fermented meats, dairy, vegetables [40]
*Lactobacillus rhamnosus*	Exopolysaccharides, lactic acid	*Salmonella* sp., spoilage yeasts	Dairy (yogurt, cheese) [41]
*Lactococcus lactis*	Nisin (bacteriocin)	Gram-positive bacteria (*Listeria* sp., *Staphylococcus* sp.)	Cheese, dairy beverages [42]
*Bifidobacterium bifidum*	Short-chain fatty acids, acetate	Enteric pathogens, spoilage bacteria	Infant formula, dairy products [43]
*Pediococcus acidilactici*	Pediocin	*Listeria monocytogenes*	Meat, fish [44]
*Saccharomyces boulardii*	Organic acids, ethanol, peptides	Spoilage fungi, bacteria	Functional beverages [45]
*Weissella cibaria*	Hydrogen peroxide, antimicrobial peptides	Gram-negative bacteria, molds	Fermented vegetables, kimchi [46]
*Bacillus subtilis*	Subtilin, surfactin	Spore-forming bacteria (*Bacillus cereus*)	Plant-based foods, soy products [47]
*Propionibacterium freudenreichii*	Propionic acid, acetic acid	Molds, yeasts	Swiss cheese, dairy [48]
*Enterococcus faecium*	Enterocins	*Listeria monocytogenes*	Meat, dairy [49]

**Table 3:** Regional Evidence on Probiotic and Postbiotic Biopreservation in Food Systems.

Region	Food Products Studied	Probiotic/Postbiotic Applied	Reported Outcomes
Africa	Fermented dairy (nunu, yogurt), fermented cereals	*Lactobacillus plantarum*, *Weissella cibaria*	Extended shelf-life, reduced Listeria and fungal spoilage [101]
Asia	Kimchi, soy sauce, natto, fermented tea	*Lactobacillus sakei*, *Bacillus subtilis* metabolites	Enhanced safety, inhibition of molds and enteric bacteria [102]
Europe	Cheese, cured meats, bakery	Nisin, pediocin, *L. lactis*	Effective Listeria control; consumer acceptance of natural labeling [103]
USA	Dairy products, ready-to-eat meats, plant-based beverages	*Lactobacillus rhamnosus*, nisin, postbiotic blends	Extended shelf-life, reduced recalls due to pathogens [104]
Latin America	Fermented maize beverages, cheese	*L. plantarum*, *Bifidobacterium* sp.	Improved microbial safety, better consumer acceptance [105]
Middle East	Yogurt, kefir, fermented vegetables	*Lactobacillus bulgaricus*, kefiran exopolysaccharides	Shelf-life extension, antifungal activity [106]

**Table 4:** Major Challenges in Probiotic and Postbiotic Biopreservation Strategies.

Challenge	Description	Example from Literature	Potential Solution/Research Direction
Stability in food matrices	Loss of activity due to pH, heat, oxygen, or storage	Nisin degradation in cheese at high pH	Encapsulation in biopolymers; stabilizers [124]
Viability of probiotics	Probiotic cells die before exerting effect	*Lactobacillus* loss during pasteurization	Freeze-drying, microencapsulation [125]
Regulatory ambiguity	No unified definition for “postbiotics”	EFSA lacks harmonized approval pathways	Codex Alimentarius-based global guidelines [126]
Cost & scalability	Industrial production costly vs. synthetic preservatives	Nisin > 10× costlier than sodium nitrite	Bioreactor optimization, precision fermentation [127]
Consumer skepticism	Concerns about safety, efficacy, and “live microbes”	Low acceptance in some Western markets	Education campaigns, labeling transparency
Antimicrobial resistance (AMR)	Risk of transferable resistance genes	*Enterococcus* strains carrying resistance	Strain screening, use of purified postbiotics [128]
Interaction with food matrices	Postbiotics less effective in fatty/complex foods	Nisin in high-fat meats loses potency	Synergistic blends (EOs, nanomaterials) [129]
Shelf-life variability	Inconsistent preservation outcomes across foods	*L. plantarum* effective in vegetables but not meat	Matrix-specific formulations [130]

## Data Availability

No new data were created or analyzed in this study. Data sharing is not applicable to this article.
